# Cardiorespiratory control and cytokine profile in response to heat stress, hypoxia, and lipopolysaccharide (LPS) exposure during early neonatal period

**DOI:** 10.14814/phy2.12688

**Published:** 2016-01-26

**Authors:** Fiona B. McDonald, Kumaran Chandrasekharan, Richard J.A. Wilson, Shabih U. Hasan

**Affiliations:** ^1^ Department of Physiology and PharmacologyHotchkiss Brain Institute & Alberta Children's Hospital Research InstituteFaculty of MedicineUniversity of CalgaryCalgaryAlbertaCanada; ^2^ Fetal and Neonatal PhysiologyDepartment of PediatricsFaculty of Medicine B271Health Sciences CenterUniversity of CalgaryCalgaryAlbertaCanada

**Keywords:** Cardiorespiratory, cytokines, hyperthermia, hypoxia, infant, infection, inflammation, neonate, SIDS

## Abstract

Sudden infant death syndrome (SIDS) is one of the most common causes of postneonatal infant mortality in the developed world. An insufficient cardiorespiratory response to multiple environmental stressors (such as prone sleeping positioning, overwrapping, and infection), during a critical period of development in a vulnerable infant, may result in SIDS. However, the effect of multiple risk factors on cardiorespiratory responses has rarely been tested experimentally. Therefore, this study aimed to quantify the independent and possible interactive effects of infection, hyperthermia, and hypoxia on cardiorespiratory control in rats during the neonatal period. We hypothesized that lipopolysaccharide (LPS) administration will negatively impact cardiorespiratory responses to increased ambient temperature and hypoxia in neonatal rats. Sprague–Dawley neonatal rat pups were studied at postnatal day 6–8. Rats were examined at an ambient temperature of 33°C or 38°C. Within each group, rats were allocated to control, saline, or LPS (200 *μ*g/kg) treatments. Cardiorespiratory and thermal responses were recorded and analyzed before, during, and after a hypoxic exposure (10% O_2_). Serum samples were taken at the end of each experiment to measure cytokine concentrations. LPS significantly increased cytokine concentrations (such as TNF *α*, IL‐1*β*, MCP‐1, and IL‐10) compared to control. Our results do not support a three‐way interaction between experimental factors on cardiorespiratory control. However, independently, heat stress decreased minute ventilation during normoxia and increased the hypoxic ventilatory response. Furthermore, LPS decreased hypoxia‐induced tachycardia. Herein, we provide an extensive serum cytokine profile under various experimental conditions and new evidence that neonatal cardiorespiratory responses are adversely affected by dual interactions of environmental stress factors.

## Introduction

Sudden infant death syndrome (SIDS) is defined as the sudden death of an infant <1 year that remains unexplained after complete autopsy and death investigation, including circumstances of death and clinical history (Willinger et al. 1991; Krous et al. 2004). Our continued reliance on a diagnosis of exclusion for this disease reflects our poor understanding of the mechanisms involved (Thach 2008). Yet, SIDS is one of the leading causes of death in infancy in the developed world, accounting for 51.6 deaths per 100,000 live births in the US in 2010 (Murphy et al. 2013) suggesting a renewed effort is warranted to understand the underlying pathology and prevent these tragic events.

A recent study by Trachtenberg and colleagues (Trachtenberg et al. 2012) highlighted that in 57% of SIDS cases there was evidence of at least two environmental risk factors (e.g., prone sleeping position, overwrapping, and exposure to pathogens) and one intrinsic risk factor (e.g., male gender, orofacial abnormality, and prenatal cigarette smoke exposure) at the time of death. These correlative data support the triple risk model for SIDS proposed by Kinney and colleagues (Filiano and Kinney 1994). According to this model, SIDS occurs in vulnerable infants who have an underlying susceptibility, when exposed to an exogenous stressor during a critical window of development. Indeed, many of the major risk factors for SIDS may be underpinned by a poor response to infection, heat stress and/or hypoxia that may interfere with control of breathing, cardiac function, arousal, and autoresuscitation. To date, few studies have systematically investigated the interactive effects of these factors on cardiorespiratory responses, which may trigger physiological distress, leading to a vicious detrimental cycle (Vege and Ole Rognum 2004). To begin to address the gaps in our knowledge, we examine both the independent and interactive effects between infection, ambient temperature, and hypoxia on cardiorespiratory control.

Epidemiologic data suggest a role for infection in the pathology of SIDS; seasonal variations are reported in the occurrence of SIDS, whereas other studies report vaccination strategies which have effectively reduced SIDS (Essery et al. 1999; Mage 2004; Vennemann et al. 2007). Furthermore, there is evidence of prior infection in at least a subset of SIDS cases as well as the presence of inflammatory mediators that have been identified on postmortem examination of these SIDS victims (Stoltenberg et al. 1992; Vege et al. 1999; Blackwell 2008; Weber et al. 2008). Nevertheless, correlative data of inflammatory mediators from SIDS victims are not yet sufficiently reliable to form a clear picture of the role of infection in SIDS due to the fact that pathology studies often occur long after death, the risk of contamination during autopsy, and the difficulty in obtaining reliable ‘control’ samples. Animal models support the hypothesis that inflammation is an important underlying mechanism of SIDS in at least a subset of cases (Morris et al. 1987; Blackwell et al. 1994; Blood‐Siegfried 2015). The use of animal models have attempted to delineate the role of inflammation and a role for IL‐1*β*‐induced prostaglandin‐mediated depression of brainstem respiratory neurons in mice (Hofstetter et al. 2007) and rat (Olsson et al. 2003) has been identified. However, further studies are needed to delineate the role of systemic inflammation on neonatal cardiorespiratory physiology and how it may alter the response to other environmental stressors (Blood‐Siegfried 2015).

Heat stress may result from overwrapping, soft underbedding, and prone sleeping position (Stanton 1984; Gilbert et al. 1992; Ponsonby et al. 1992; Tuffnell et al. 1995; Fleming et al. 1996; Williams et al. 1996; Baddock et al. 2004) and indeed, heat stress may exacerbate the effects of infection (Gilbert et al. 1992). In addition, animal data demonstrate that heat stress negatively affects autoresuscitation in rats and mice (Kahraman and Thach 2004; Serdarevich and Fewell 1999; Tomimatsu et al. 2003). No animal studies to date have addressed the interaction between heat stress and inflammation on neonatal breathing.

One of the long‐standing hypotheses in SIDS is that an ineffective response to a hypoxic event may be the final trigger for SIDS (Vege et al. 1994; Opdal et al. 1998). While in a prone sleeping position, or if the head becomes covered, infants may be exposed to severe rebreathing of expired gases in which the O_2_ is dramatically lowered and the CO_2_ is raised. The size of the response to hypercapnic hypoxia is blunted and the response rate to this stimulus is delayed in early life (Sovik and Lossius 2004). Infants, especially those premature (a risk factor for SIDS), have abnormal arousal and diminished ventilatory responses to hypoxia and hypercarbia (Hunt 1981; van der Hal et al. 1985). Animal data provide evidence that hyperthermia can prolong the laryngeal chemoreflex‐induced apnea (Xia et al. 2008) and hyperthermia with/without hypoxia can result in a failure of eupneic breathing in young rat pups (Pendlebury et al. 2008; Ferng and Fregosi 2015). Moreover, when hyperthermia is combined with anoxia, the time to last gasp decreases along with the survival rate and ability to auto‐resuscitate during anoxic challenge (97% N_2_ and 3% CO_2_) (Kahraman and Thach 2004; Serdarevich and Fewell 1999). The physiological response to hypoxia under a hyperthermic and inflammatory state during development remains unknown.

Evidence suggests that the multiple exogenous stressors (e.g., inflammation, heat stress, and hypoxia) may interact to significantly alter normal cardiorespiratory output, resulting in distress of an infant. Neonates in this environment must process multiple sensory signals to mount the most appropriate response trying to balance between O_2_ requirements, acid‐base balance, heat control, and inflammatory stimuli. There is a need to use an animal model as a tool to explain how multiple environmental risk factors could contribute to the cause of death. Therefore, this study aimed to quantify the independent and possible interactive effects of infection, hyperthermia, and hypoxia on cardiorespiratory control in rats during the neonatal period. We hypothesized that LPS administration will negatively impact cardiorespiratory responses to increased ambient temperature and hypoxia in neonatal rats.

## Methods

### Ethical approval

All studies were performed in accordance with The Canadian Council on Animal Care Guidelines and were approved locally by the Animal Care Committee of the Cumming School of Medicine, University of Calgary, Canada.

### Animals

Pregnancy was confirmed by observation of vaginal plug after overnight mating. Rats were maintained in standard housing conditions with access to standard chow and water ad libitum. The offspring were studied between postnatal days (P) 6–8 and were reared with their respective mothers until the start of each experimental protocol. To investigate the relationship between rectal (T_R_), body surface (T_BS_), and ambient temperature (T_a_), six P7 animals were studied (3 litters). To investigate the cardiorespiratory response to infection, hyperthermia, and hypoxia, only one rat per litter was assigned to one of the six treatment groups. A control (*n* = 10), saline (*n* = 11), and LPS group (*n* = 11) were studied at T_a_ of 33°C. Similarly, a control (*n* = 12), saline (*n* = 12), and LPS group (*n* = 10) were studied at a higher ambient temperature of 38°C. The T_a_ 33°C used in this study is within the thermoneutral range for P6‐8 rat pups (Conklin and Heggeness 1971; Malik and Fewell 2003). T_a_ 38°C was chosen as a high environmental temperature, 1 degree above the adult core temperature of most mammals. LPS was administered at dose of 200 *μ*g/kg, which has previously been used as a surrogate for infection (Heida et al. 2004). Saline was given at a volume equal to that of LPS dose‐volume. Both saline and LPS were administered intraperitoneal (IP) two hours prior to start of the protocol. The two‐hour incubation period for LPS was chosen so that the experiments could be performed during the time‐frame of two to three hours post injection as a previous study demonstrated the maximum body temperature increase during this window (Heida et al. 2004).

### Correlation between rectal, body surface, and ambient environmental temperature

A fine thermocouple (Sper Scientific, Scottsdale, AZ) was attached to a bandage (Co‐Flex, Andover, MA), and secured on the ventral surface of the chest to measure T_BS_. A second thermocouple probe was placed rectally to measure core temperature. The pup was then placed in a custom‐made water‐jacketed glass vessel (volume ~60 mL), through which there was continuous flow of water from a heated water bath to ensure a stable environmental temperature for at least 10 min. T_a_ was measured using a third thermocouple probe inside the glass chamber. Temperature inside the chamber was increased from 30°C to 38°C and corresponding body T_BS_ and T_R_ were recorded and plotted as a scatter plot with a best fit linear regression line.

### Head‐out plethsymography

The head‐out plethysmography technique, which is used to assess ventilation in unanesthetized neonatal rodents was previously described by Cummings et al. (1985). In brief, the apparatus consisted of two custom‐made chambers; one for the head and another for the body. The nose and mouth of the pup were sealed into a head‐chamber with polyether impression material (3 M Impregum F, St. Paul, MN). The rat pup was then placed in a custom‐made water‐jacketed glass vessel as described above. T_a_ in this chamber was monitored using a fine thermocouple (Sper Scientific, Scottsdale, AZ). The nose and mouth were then exposed to a steady gas flow (497 mL/min) of 21% O_2_ balance N_2_. The gas flow was generated by a vacuum pump downstream of the head‐chamber (AEI Technologies, Naperville, IL). A pneumotach (Omega Engineering, Stamford, CT) attached to the head‐chamber allowed direct measurement of tidal volume (*V*_T_); this device was calibrated using rapid air injections (20 *μ*L). The flow signal was amplified, filtered (Brownlee Precision model 440; low pass 20 Hz; high pass 0.5 Hz), digitized (Axon Instruments Digidata 1322A; sample rate 1 kHz), and recorded in volts for later analysis (AxoScope 9). The flow tracing was later integrated and peak height was used to determine *V*_T_ and frequency (*f*_*R*_ *; breaths per minute*) to allow minute ventilation (${\dot{V}}_{E}$) to be calculated (LabChart 7).

### Experimental protocol

On the day of the experiment, each pup was weighed and a bandage fitted around the chest of the pup to which surface electrodes for electrocardiographic (ECG) measurements and a fine thermocouple (Sper Scientific, Scottsdale, AZ) was attached for continuous recording of the T_BS_. T_BS_ was measured instead of T_R_ to minimize animal stress. The head and mouth of the animal were then placed in the head‐chamber and sealed with polyether impression material around the nitrile rubber (Microflex, Reno, NV). The animal was then placed in the body chamber and was allowed time to acclimatize to the environmental temperature (33°C or 38°C). Thereafter, a 5‐min baseline period at 21% FiO_2_, a 5‐min hypoxic period at 10% FiO_2_ and a 5‐min posthypoxic recovery period at 21% FiO_2_ were recorded. The rats were then euthanized as recommended by the Animal Care Centre SOP E1 for rodent euthanasia. A blood sample was collected postmortem for cytokine analysis.

### Data analysis

Breathing and ECG data were analyzed using LabChart 7 (AD Instruments Inc, Colorado Springs, CO), a digital strip chart recorder. The last minute of each period (baseline, hypoxia, and recovery) was used to assess quiet breathing. *V*_*T*_, *f*_R_, ${\dot{V}}_{E}$, coefficient of breathing frequency variability (CV*f*_R_), and heart rate (HR) were assessed. ${\dot{V}}_{E}$ (mL min^−1^ g^−1^) was calculated as the product of the *f*_R_ (breaths min^−1^) and the *V*_*T*_ (mL g^−1^). Integration of the flow changes associated with respiratory activity was used to calculate the *V*_*T*_, whereas the *f*_R_ was obtained directly from the breathing tracings. CV*f*_R_ was calculated as the ratio of the standard deviation and the mean frequency (SD/mean*f*_R_).

All data are presented as mean ± SD unless otherwise stated. Body mass and postnatal age data were analyzed by one‐way analysis of variance (ANOVA) (GraphPad Prism 6) to assess any differences between groups prior to experimental intervention. To test the effects of individual experimental factors and the interactions of multiple experimental factors on body surface temperature, V_T_, *f*_R_, ${\dot{V}}_{E}$, CV*f*_R_, and HR, three‐way ANOVAs were carried out on the absolute data. The three independent factors were; gas period (three levels; normoxia, hypoxia, and posthypoxia recovery), ambient temperature (two levels; 33 and 38°C), and LPS injection (three levels; control, saline, and LPS).

To further investigate where the difference laid, additional two‐way ANOVAs were used to separately compare LPS and temperature responses during the baseline, hypoxic (% increase from baseline), and posthypoxic periods (% of the baseline). The two independent factors were; ambient temperature (two levels; 33 and 38°C) and LPS injection (three levels; control, saline, and LPS). The Sidaks post hoc test was applied when ANOVA revealed a significant factorial difference. A *P* ≤ 0.05 was considered statistically significant.

### Cytokine analysis

Blood was collected, spun down and serum stored at ≤ −20°C. Samples were diluted twofold and samples analyzed in duplicate. Twenty‐seven cytokine/chemokine biomarkers were quantified simultaneously by Eve Technologies Corp, Calgary, AB, Canada using a Discovery Assay^®^ (Rat Cytokine Array/Chemokine Array 27‐Plex). The multiplex assay was performed using the Bio‐Plex^™^ 200 system (Bio‐Rad Laboratories, Inc., Hercules, CA), and a Milliplex rat cytokine kit (Millipore, St. Charles, MO) according to the manufacturer's protocol. The 27‐Plex consisted of Tumor Necrosis Factor alpha (TNF*α*), Interleukin‐1*α* (IL‐1*α*), IL‐1*β*, IL‐18, IL‐12(p70), IL‐6, IL‐17A, Interferon‐*γ* (IFN*γ*), Leptin, IL‐2, IL‐5, IL‐4, IL‐13, IL‐10, Macrophage Inflammatory Protein 1 alpha (MIP‐1*α*), Macrophage Inflammatory Protein 2 (MIP‐2), Regulated on Activation, Normal T Expressed and Secreted (RANTES), Monocyte Chemotactic Protein 1 (MCP‐1), Eotaxin, IFN‐gamma‐inducible protein 10 (IP‐10), human Growth‐Regulated Oncogene/Keratinocyte Chemoattractant/Cytokine‐Induced Neutrophil Chemoattractant 1 (GRO/KC/CINC‐1), Lipopolysaccharide‐Induced CXC chemokine (LIX), Fractalkine, Epidermal Growth Factor (EGF), Vascular Endothelial Growth Factor (VEGF), Granulocyte‐Colony Stimulating Factor (G‐CSF), and Granulocyte‐Macrophage Colony‐Stimulating Factor (GM‐CSF). The sensitivities of these assays ranged from 0.1to 15.7 pg mL^−1^. Data were analyzed initially for outliers using Grubbs (remove only one outlier 0.05 certainty). Cleaned data were then analyzed by two‐way ANOVA (temperature × LPS).

## Results

### Postnatal age and body mass

The body mass and postnatal ages were similar across the experimental groups (Table 1).

**Table 1:** Data (mean ± SD) for body mass (g) and postnatal age (day) of rat pups in each group prior to any experimental treatment (one‐way ANOVA)

	T_a_ 33°C			T_a_ 38°C			One‐way ANOVA
	Control	Saline	LPS	Control	Saline	LPS	
*n*	10	11	11	12	12	10	
Body Mass (g)	16.27 ± 2.61	17.14 ± 2.10	17.26 ± 2.99	17.25 ± 2.87	17.3 ± 2.2.4	16.02 ± 2.08	*P* = 0.77
Postnatal age (day)	6.5 ± 0.7	7 ± 0.7	7 ± 0.9	7 ± 1.0	7 ± 0.7	7 ± 0.8	*P* = 0.88

Body mass and age were similar across groups. T_a_‐ Ambient temperature.

### Body surface temperature

**Figure 1:**
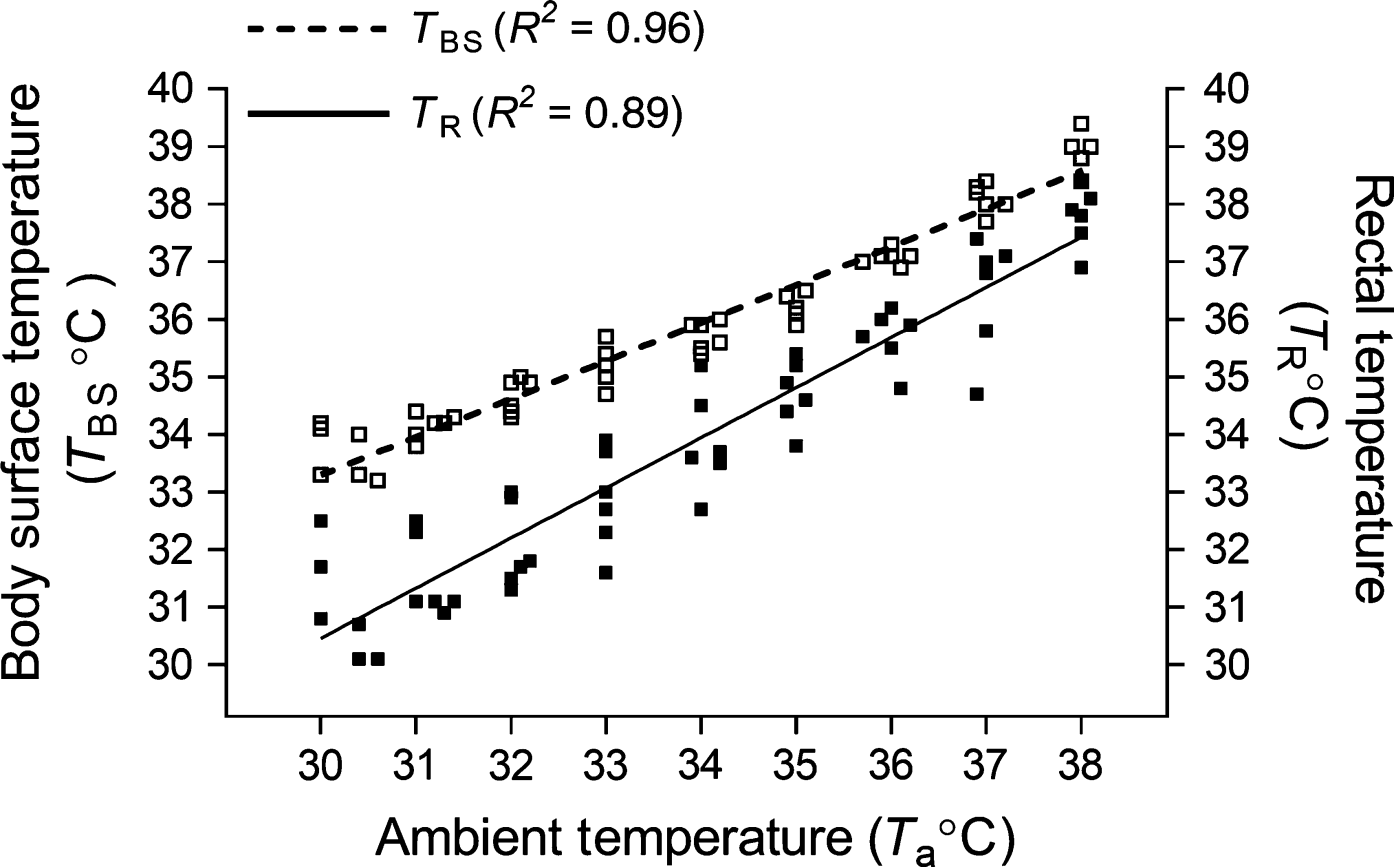
Scatter plot with linear regression line showing correlation between ambient (T_a_), body surface (T_BS_), and rectal temperature (T_R_) in postnatal day 7 rat pup (*n* = 3; 2 pups from 3 separate litters). The relationship between T_BS_ and T_R_ at a given ambient temperature is described by relating both equations of the line (Y = 0.6597*X + 13.51 and Y = 0.8703*X + 4.354), *Y*_Rectal_ *= *1.32*∙Y*_Body surface_ *‐*13.44. The core temperature closely reflects ambient temperature.

The relationship between T_BS_, T_R_, and T_a_ is demonstrated in Figure 1; rectal (core) temperature reflects ambient temperature.

**Figure 2:**
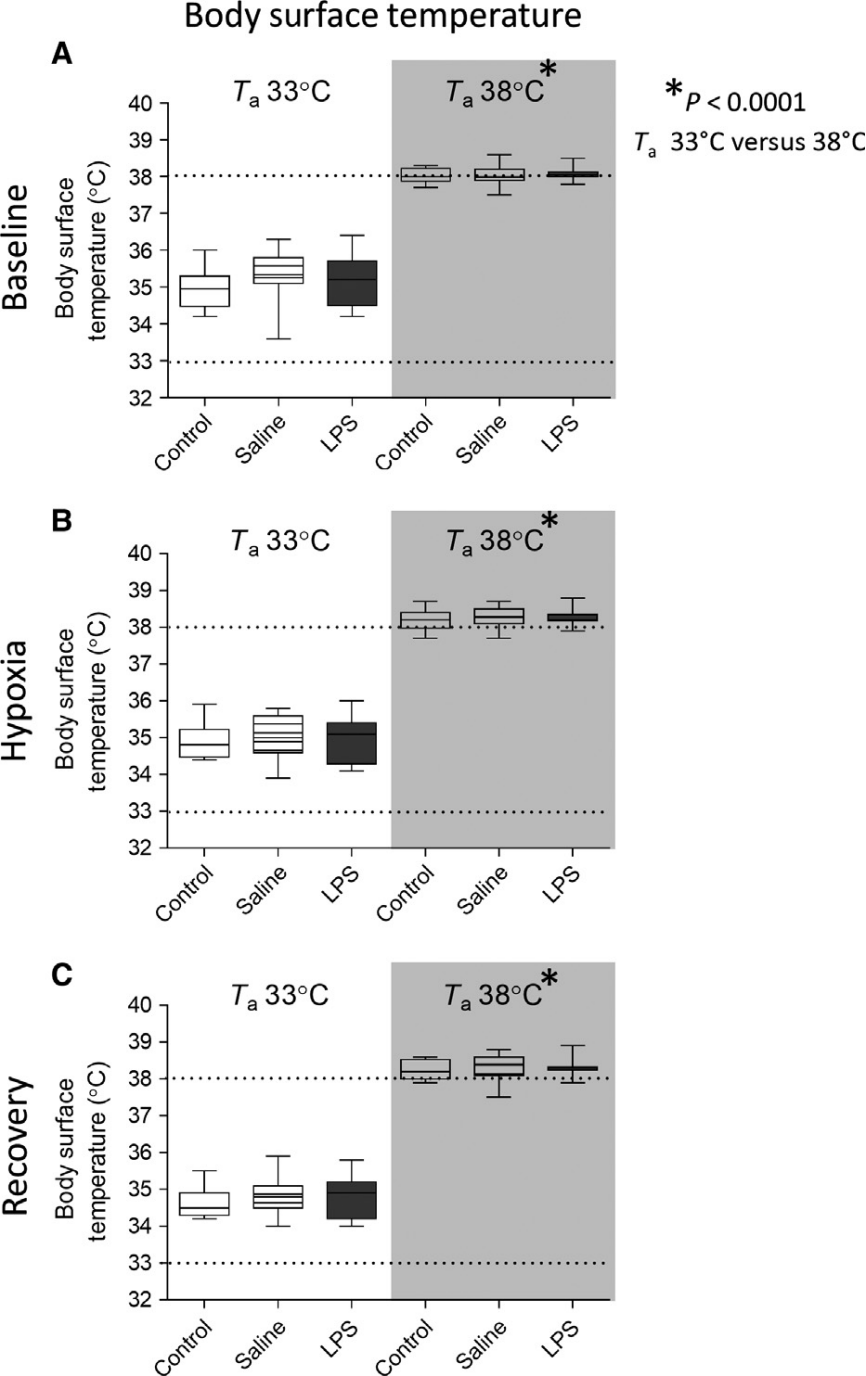
Data (box plot illustrating minimum, first quartile, median, third quartile, and maximum; *n* = 10–11 per group) for rat pup body surface temperature under ambient temperature (T_a_) of 33°C and 38°C during baseline (A), hypoxic (B), and posthypoxic recovery (C) periods in control, saline, and LPS treatment groups. The body surface temperatures were higher in animals at ambient temperature of T_a_38°C versus T_a_33°C. LPS and hypoxia did not significantly change body surface temperature (three‐way analysis of variance (ANOVA); temperature x gas period x LPS).

Experimental data for T_BS_ at T_a_ 33 and 38°C recorded at baseline, hypoxia, and recovery in control, saline, and LPS treatment groups are presented in Figure 2. Three‐factor analysis (gas period × temperature × LPS) demonstrated an increase in T_BS_ with higher T_a_ (*P* < 0.0001). LPS treatment did not affect T_BS_ under any experimental condition (*P* = 0.18).

### Breathing variables

Three‐factor (gas period × temperature × LPS) ANOVA was performed separately on V_T_, *f*_R_, and${\dot{V}}_{E}$. V_T_, *f*_R_, and ${\dot{V}}_{E}$ were significantly different between gas periods (*P* < 0.0001); hypoxia increased V_T_, *f*_R_, and ${\dot{V}}_{E}$ (P < 0.05; post hoc test), whereas *f*_R_ and ${\dot{V}}_{E}$ during the posthypoxic recovery period were lower than the baseline and hypoxic values (P < 0.05; post hoc test). Posthypoxic V_T_ was not different from baseline values. Furthermore, there was an interaction between temperature and gas period on V_T_, *f*_R_, and ${\dot{V}}_{E}$ (*P* < 0.05) and between temperature and LPS on V_T_ only.

**Figure 3:**
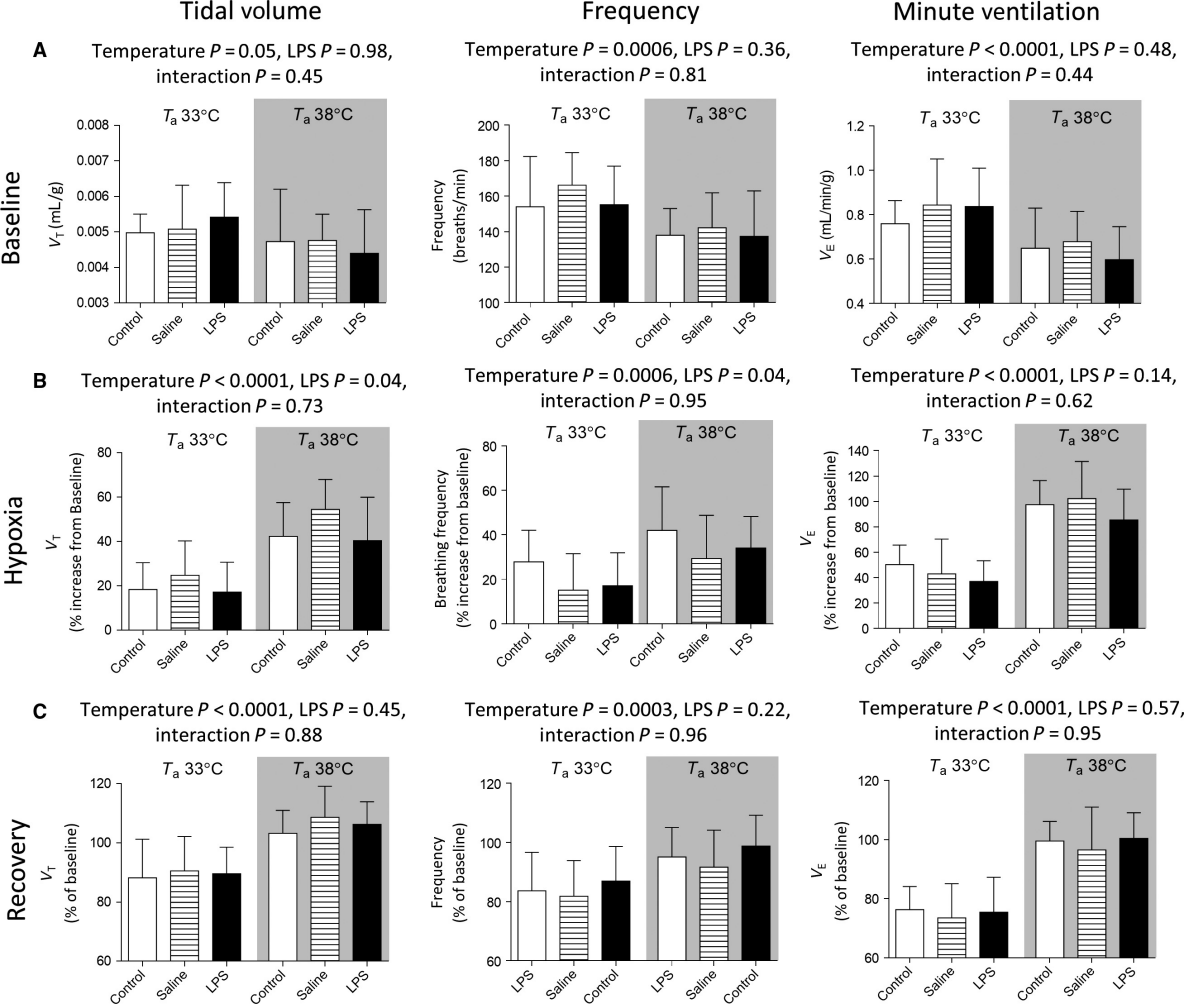
Baseline (A), hypoxia (B), and posthypoxia recovery (C) data (mean ± SD;* n* = 10–12 per group) for tidal volume (V_T_; normalized to body weight), frequency (*f*_R_; breaths per minute), and minute ventilation (${\dot{V}}_{E}$= V_T_∙*f*_R_). The hypoxic response is expressed as percentage increase from baseline. Posthypoxic recovery is expressed as percentage of the initial baseline values. V_T__,_ *f*_R__,_ and ${\dot{V}}_{E}$ were lower under T_a_38°C versus T_a_33°C at baseline (A). Hypoxic ventilatory response (V_T__,_ *f*_R__,_ ${\dot{V}}_{E}$) was higher at T_a_38°C versus T_a_33°C (B). Posthypoxic ventilatory decline (V_T__,_ *f*_R__,_ ${\dot{V}}_{E}$) was greater at T_a_33°C versus T_a_38°C (C). Data were analyzed using two‐way ANOVA (LPS X Temperature). T_a_‐ Ambient temperature.

Two‐factor (temperature x LPS) ANOVA's were performed to assess the ventilatory responses during each period (Fig. 3A–C). Under baseline (normoxic) conditions, V_T_, *f*_R_, and ${\dot{V}}_{E}$were suppressed by the higher ambient temperature (*P* = 0.05; *P* = 0.0006; *P* < 0.0001, respectively; Fig. 3A). There was no effect of LPS treatment on baseline breathing responses and no interaction between temperature and LPS was observed. V_T_, *f*_R_, and ${\dot{V}}_{E}$ under hypoxic conditions are expressed as percent increase from the baseline values (Fig. 3B). There was a more pronounced hypoxic ventilatory response at T_a_ 33°C versus 38°C (V_T_ *P* < 0.0001, *f*_R_ *P* = 0.0006, ${\dot{V}}_{E}$ *P* < 0.0001, respectively). LPS as a factor was significant for V_T_ and *f*_R_ (*P* = 0.04, *P* = 0.04, respectively), however, post hoc tests did not illustrate any independent changes due to LPS treatment (Fig. 3B). V_T_, *f*_R_, and ${\dot{V}}_{E}$ during the posthypoxic recovery period are expressed as percent of baseline values (Fig. 3C). Posthypoxic ventilatory decline (V_T,_ *f*_*R*_ *,* and${\dot{V}}_{E}$) was greater at T_a_ 33°C compared to 38°C (*P* < 0.0001, *P* = 0.0003, *P* < 0.0001, respectively). There was no significant effect of LPS by itself or in combination with heat stress.

### Coefficient of variance of breathing frequency

**Figure 4:**
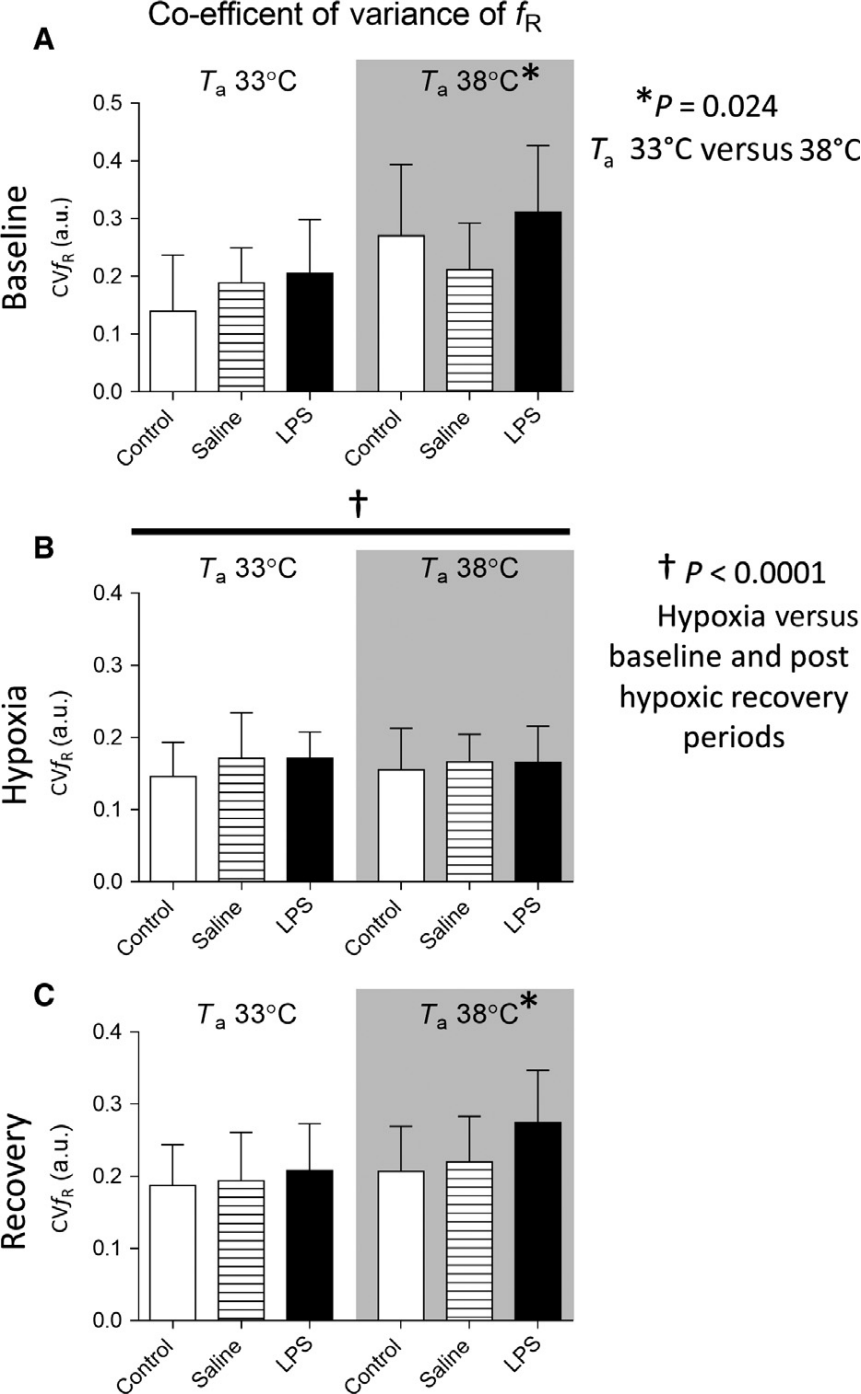
Coefficient of variance of breathing frequency (CV *f*_R_) (mean ± SD;* n* = 10–12 per group) during baseline (A), hypoxic (B), and posthypoxic recovery (C) periods in neonatal rat pup. Data were analyzed by three‐way ANOVA. The CV *f*_R_ was increased in high ambient temperatures (T_a_38°C versus T_a_33°C) in baseline and posthypoxic recovery periods (A and C). CV *f*_R_ was lower in hypoxia compared with the baseline and posthypoxic periods. T_a_ – Ambient temperature.

CV*f*_R_ was increased at higher ambient temperature in baseline and posthypoxic recovery periods (T_a_ 38°C versus 33°; *P* = 0.02; Fig. 4). Variability in breathing frequency was lower in hypoxia compared to baseline and posthypoxic recovery periods (*P* < 0.0001). LPS did not alter CV*f*_R_ (*P* = 0.12).

### Heart rate

Three‐factor (gas period × temperature × LPS) ANOVA was performed on absolute HR. HR was higher during hypoxia compared with the baseline and posthypoxic recovery values at both ambient temperatures; post hoc *P* < 0.001). Such increase was more pronounced under higher ambient temperature (T_a_ 38°C versus 33°C; *P* < 0.0001).

**Figure 5:**
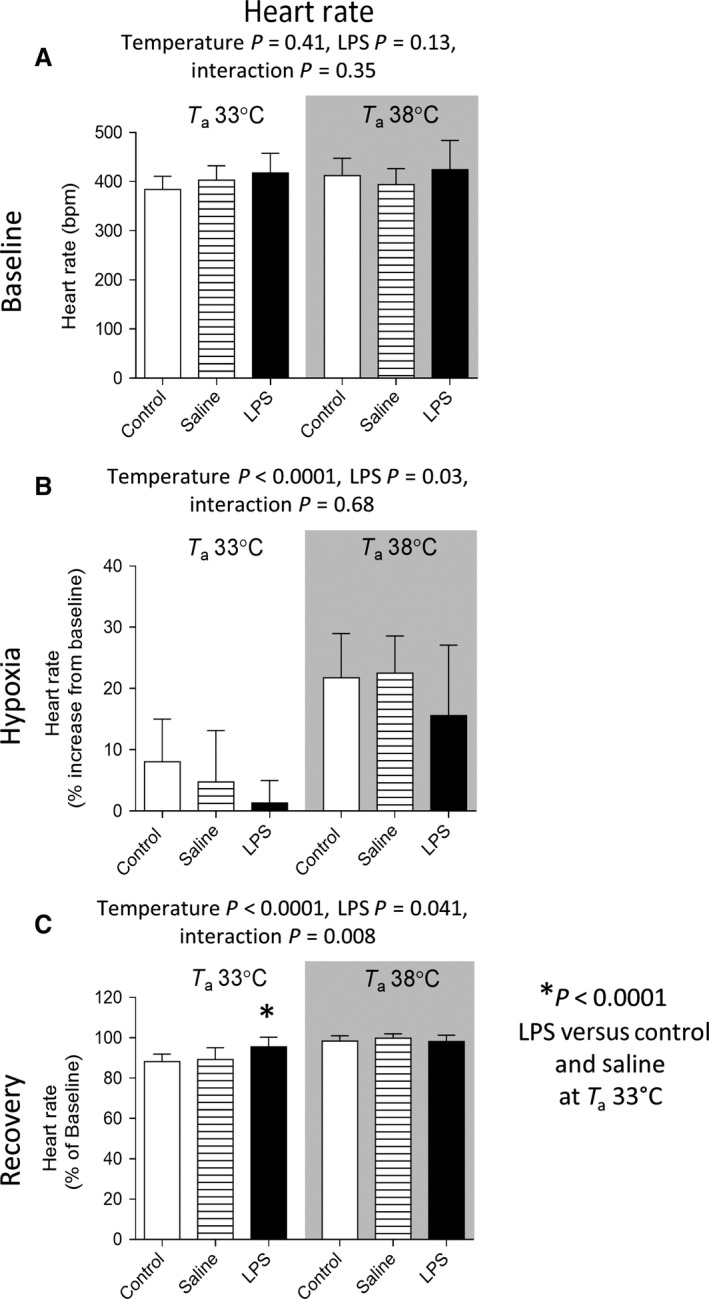
Group data (mean ± SD;* n* = 8–10 per group) for heart rate during baseline (A), hypoxic (B), and posthypoxic recovery (C) periods. Hypoxic response is expressed as percentage change from baseline. Posthypoxic recovery is expressed as percentage of baseline. Data were analyzed by two‐way ANOVA and *P*‐values are reported in the figure. HR was blunted by LPS under both low and high ambient temperatures during hypoxia (B). During the posthypoxic recovery period, HR of animals at T_a_ 33°C took longer to return to the baseline values compared to those at higher T_a_ 38°C, however, the lower HR was not observed in animals treated with LPS at T_a_ 33°C (C). T_a_ – Ambient temperature.

Two‐factor (temperature × LPS) ANOVA revealed that HR was blunted by LPS under both low and high ambient temperatures during hypoxia (*P* = 0.03; Fig. 5B). During the posthypoxic recovery period, HR of animals at T_a_ 33°C took longer to return to the baseline values compared to those at higher T_a_ 38°C (*P* < 0.0001; Fig. 5C), however, the lower HR was not observed in animals treated with LPS at T_a_ 33°C (*P* = 0.008).

### Cytokines

Detailed serum cytokine concentrations (pg mL^−1^) are given in Table 2, alongside the two‐way (temperature × LPS) ANOVA results for each cytokine. Briefly, TNF*α*, IL‐1*β*, IL‐12, IFN*γ,* and IL‐5 increased in the LPS‐treated group compared with control and saline groups (*P* ≤ 0.05; Table 2). Additionally, the anti‐inflammatory cytokine IL‐10 increased with LPS *(P < *0.0001*;* Table 2). Chemokine levels of MIP‐1*α*, MIP‐2 MCP‐1, IP‐10, GRO/KC, and fractalkine were also elevated (*P* < 0.05), whereas RANTES levels were lower in the LPS‐treated animals (*P < *0.02). Furthermore, IFN*γ*, IL‐5, and fractalkine increased under higher ambient temperature (*P* = 0.05, *P* = 0.01, *P* = 0.03, respectively). Also, an interaction was observed between LPS and higher T_a_ for IFN*γ*, G‐CSF, and GM‐CSF (*P* = 0.05, *P* = 0.05, *P* = 0.01; Table 2).

**Table 2:** Serum cytokines concentrations (pg/ml) in control, saline and LPS treated rat pups

	T_a_33°C			T_a_38°C			Two‐way ANOVA		
	Control	Saline	LPS	Control	Saline	LPS	LPSx Temperature	LPS	Temperature
	Concentration pg mL^−1^						*P*		
Pro‐inflammatory Cytokines									
TNFα	28.5±6.8	23.9±4.2	295.1±266.6	35.3±16.2	28.8±6.9	558.1±640.9	0.36	0.0004*	0.26
IL‐lα	424±567.6	460.5±640.2	138.2±106.6	1050±934.2	1115±1192	58.5±98.9	0.44	0.07	0.11
IL‐1β	50.7±35.9	31.4±21.2	286±461.7	88.9±27.8	51.7±28.5	366.4±470.2	0.96	0.0176*	0.58
IL‐18	6818±4911	6258±3346	9005±8404	7244±7778	4316±1350	5002±2125	0.59	0.65	0.30
IL‐12(p70)	643.6±179.3	485.8±144.7	782.6±345.2	617.8±185.7	736.2±155.8	836.9±148.8	0.22	0.05^†^	0.18
IL‐6	558.3±363.9	468.8±333.6	1228±826.1	1336±833.1	944±719.8	14886±23428	0.10	0.06	0.07
IL‐17A	35.3±9.0	33.2±14.6	44.9±17.1	51.8±20.0	37.5±9.6	49.3±16.8	0.45	0.12	0.08
IFNy	197.2±96.8	171.8±68.8	311.6±147.5	228.2±30.9	201.6±57.0	1353±1591	0.05*	0.012*	0.05*
Leptin	23517±18413	15291±6879	11878±8276	22130±13870	19282±10200	11713±3499	0.83	0.09	0.84
IL‐2	174.7±64.0	134.9±73.1	195.2±132.9	209.4±29.9	232.6±109.1	228.9±70.4	0.57	0.72	0.06
IL‐5	109.7±34.79	88.61±12.12	155.2±39.08	140.9±38.59	124.1±30.91	175.3±26.98	0.83	0.0003*	0.01*
Anti‐inflammatory									
IL‐4	7.833±4.022	5.505±1.313	17.7±13.92	12.82±5.055	8.633±4.605	9.653±12.165	0.08	0.10	0.99
IL‐13	39.4±15.2	42.1±17.6	49.7±30.7	49.3±17.6	50.9±18.5	42.7±10.0	0.50	0.95	0.54
IL‐10	106.8±50.3	71.0±22.23	799±598.9	145.3±43.85	80.9±46.02	1027±1088	0.82	<0.0001*	0.53
Chemokines CC									
MlP‐lα	53.2±7.3	47.02±6.2	247.3±69.4	76.28±14.9	54.44±8.6	221.8±60.7	0.78	<0.0001*	0.95
MIP‐2	158.3±17.3	166.1±20.4	1530±510.6	259.8±33.3	183.6±26.2	3450±2283	0.38	0.0058*	0.27
RANTES	9223+1805	7531±1497	4726±1014	12051±3418	8656±2204	4548±489.9	0.76	0.0203*	0.45
MCP‐1	4856+1150	3028±377.6	32924±10469	10541±4812	6969±2574	37003±17495	0.99	0.0007*	0.47
Eotaxin	16.5+2.3	13.6±3.3	21.2±2.3	25.2±3.2	18.5±2.4	17.7±1.4	0.10	0.17	0.14
CXC									
IP‐10	283.9±91.9	238.3±53.8	1147±646.2	349±111.3	323.2±111.4	839.1±437	0.25	<0.0001*	0.61
GRO/KC	87.7±152.2	99.8±201.8	1494±1001	261.9±193.4	148.4±173.7	871±968.5	0.18	<0.0001*	0.48
LIX	5663±418.2	5462±356.8	4863±778.9	5667±428.8	5403±470.8	5930±1661	0.69	0.91	0.56
CX3C									
Fractalkine	203.5 ±19.8	201.1±24.8	518.3±81.4	318.6±44.2	283.1±45.5	863.8±283.6	0.37	<0.0001*	0.03*
Growth factors									
EGF	33.3±31.9	16.7±22.5	24.5±130.8	44.4±35.9	70.3±56.1	16.0±17.2	0.08	0.22	0.10
VEGF	209.6±50.7	185.4±52.3	256.6±151	246.3±19.3	227.1±52.8	212±42.2	0.31	0.62	0.65
Gylcoproteins									
G‐CSF	67.1±23.1	101.1±75.2	99±60.4	150.6±82.3	72.1±25.2	62.6 ± 8.7	0.05*	0.22	0.44
GM‐CSF	67.12±23.1	101.1±75.2	99±60.4	150.6±82.3	72.1±25.2	62.6 ± 8.7	0.01*	0.39	0.73

Group data (mean ± SD; *n* = 5–9 per group) for serum cytokine concentrations (pg mL−1) measured using the Rat Cytokine Array/ Chemokine Array 27‐Plex. Data were analyzed initially for outliers using Grubbs (remove only one outlier 0.05 certainty). Cleaned data was analyzed for each cytokine using two‐way ANOVA and *P* values are presented in the table; *P ≤ 0.05. T_a_ ‐ Ambient temperature.

## Discussion

This study aimed to test the hypothesis that aberrant cardiorespiratory responses may result from an interaction between inflammation, heat stress, and hypoxia. We designed the study to examine the individual and combined effects of LPS, high ambient temperature, and hypoxia, on cytokine profile and the cardiorespiratory responses in the neonatal rat. We report a number of key findings: (1) LPS administration increases many proinflammatory cytokines in 1‐week‐old pups but did not elicit febrile response, (2) heat stress reduced baseline breathing and augmented the hypoxia‐induced cardiorespiratory responses, and (3) treatment of rat pups with LPS blunted the hypoxia‐related tachycardia observed in control animals but exerted little effect on breathing responses in neonatal rat pups.

### LPS‐induced cytokine expression and thermal response

Limited data are available on LPS‐induced cytokine expression in neonatal rats. Thus, one of the objectives of this study was to establish the complement of pleiotropic polypeptides (cytokines) coordinating the inflammatory response in LPS‐treated neonatal rats. Potent proinflammatory cytokines were upregulated such as TNF*α*, IL‐1*β* among others. Conversely, LPS increased serum levels of IL‐10, an anti‐inflammatory cytokine capable of reducing TNF*α*, IL‐1*β,* and IL‐6 levels (Hedi and Norbert 2004). The extent and duration of the LPS‐induced inflammation is determined by the balance between pro‐ and anti‐inflammatory cytokines. LPS administered IP resulted in a robust inflammatory response in our neonatal rats as evidenced by the upregulation of numerous cytokines including IP‐10, a reported bio‐marker of neonatal bacterial infection (Wagner et al. 2011). We provide new evidence that heat stress can alter the cytokine response. In our study, we observed an increase in IFNy, IL‐5, and fractalkine levels under the higher ambient temperature. Moreover, heat stress interacts with LPS leading to increased serum concentrations of IFNy, G‐CSF, and GM‐CSF. We speculate that the cardiorespiratory responses to environmental challenges, reported within this study may be mediated, in part, by upregulation of cytokines.

Despite the LPS‐induced upregulation of cytokines, there was no evidence of a concurrent fever, which might be due to the age of our animals and/or experimental conditions (Cao and Watanabe 1997; Fraifeld and Kaplanski 1998; Heida et al. 2004). Fever does not generally occur in response to infection in infants less than 1 month; in fact, these neonates often have a hypothermic response to infection (Hofer et al. 2012). In our study, pyrogenic cytokines such as TNF*α*, IL‐1, and IFNy were upregulated in LPS‐treated pups, but one of the other powerful pyrogenic cytokines, IL‐6 was not. On one hand, the absence of fever may indicate a poor central inflammatory response. However, on the other hand, the absence of a fever allowed us to interpret the data without accounting for change in thermoregulatory set point and may be more representative of the mild infection reported in SIDS cases. Therefore, in this study, an increase in body temperature by raising the ambient temperature, better models external warming associated with SIDS (such as over wrapping and higher room temperatures) and not fever. Fever is the biological resetting of thermoregulatory set point to a higher temperature, whereas hyperthermia is a rise in body temperature beyond the thermoregulatory set point.

### Effect of LPS on cardiorespiratory variables under normoxia and hypoxia

In this study, LPS did not alter ventilatory variables during normoxia, which is in agreement with previous observations in piglets (McDeigan et al. 2003). In contrast, studies by Olsson et al. demonstrated respiratory depression in response to IL‐1*β*. The effect of IL‐1*β* on eupneic breathing was more deleterious compared with LPS in the same study; LPS was reported to cause a small (but not significant) decrease in breathing frequency and no change in ${\dot{V}}_{E}$ (Olsson et al. 2003). Furthermore, there were a number of methodological differences in their study compared to ours, including repeated i.p. injections and lower temperature conditions. Furthermore, LPS treatment did not alter HR at baseline, although significant tachycardia has been reported following extremely high doses (1 mg kg^−1^, 25 mg kg^−1^) of LPS in neonatal rats and mice, respectively (Mukherjee et al. 2010; Yang et al. 2010). Our data suggest that mild LPS‐induced inflammatory response, which floods the system with numerous pro‐ and anti‐inflammatory cytokines, attenuates the severity of independent, unopposed actions of certain cytokines such as IL‐1*β* (Olsson et al. 2003). While we recognize the need to understand the role of individual cytokines, we believe in the context of SIDS, it is vital that we investigate the effects of a global inflammatory response on neonatal cardiorespiratory control.

Significant maturation of the respiratory control system occurs during postnatal life as it transitions from intrauterine life, resetting its chemoreceptors (Carroll and Kim 2013). Therefore, the neonatal period can be a vulnerable time for an infant in regards to sensing blood‐gas changes, alongside limited locomotor skills to reposition the head if the face becomes covered until approx. 32 weeks of age (Darrah and Bartlett 2013). In this study, neonatal rats had marked hyperpnea on exposure to a short, mild hypoxic challenge. The hypoxic response in mammals is biphasic, with an initial increase in pulmonary ventilation mediated by the carotid body, followed by an inhibitory phase mediated by central release of adenosine (Lahiri and DeLaney 1975; Yan et al. 1995; Teppema and Dahan 2010). The initial phase increases with maturation, whereas the secondary phase is most apparent in the first few days of life in the rat and with increasing severity of hypoxia (Eden and Hanson 1987; Teppema and Dahan 2010). By 1 week, the second (inhibitory) phase of the hypoxic response is much smaller in rats (Eden and Hanson 1987). In the presence of LPS, there was a trend toward reduced hypoxic ventilatory response (HVR); however, it did not reach significance. Previous studies have shown that LPS can depress the HVR but through different routes of administration (intratracheal or intravenous), which might result in higher levels of cytokines communication with the brain. Studies have suggested that these effects are mediated at the level of the brainstem (McDeigan et al. 2003; Balan et al. 2011; Siljehav et al. 2012). In contrast, Ladino et al. (2007) reported that LPS‐induced depression in early, but not in late HVR, in juvenile rats, suggesting an effect at the level of the peripheral oxygen chemoreceptors (Zhang et al. 2007; Fernández et al. 2008; Master et al. 2015).

The hypoxia‐induced tachycardia observed in neonatal rat pups in our study corroborates previous observations in adult rats (Marshall and Metcalfe 1990; Rohlicek et al. 2002) and is likely mediated through Hering–Breuer reflex and aortic body baroreceptors (O'Regan and Majcherczyk 1982; Kato et al. 1988). The percentage change from baseline heart rate during LPS administration was attenuated during hypoxia. Under clinical and experimental conditions, heart rate increases during an inflammatory process for several reasons including vasodilation (Rabuel and Mebazaa 2006) and poor myocardial contractility (Parrillo et al. 1985; Merx and Weber 2007). It is, therefore, plausible that inflammatory states attenuate the hypoxia‐associated increase in heart rate observed in control animals due to myocardial impairment. The blunted hypoxic‐induced gain in heart rate may be a maladaptive response leading to hypoperfusion and ischemic hypoxia in the absence of vascular compensation.

### Effect of heat stress on cardiorespiratory variables under normoxia and hypoxia

Neonatal infants do not regulate their temperature well, with large body surface to volume ratio similar to neonatal rats; therefore an appropriate environmental temperature is important for homeostasis. We report a decrease in ${\dot{V}}_{E}$ primarily through a reduction in *f*_R_ under higher ambient temperature. Although previous studies in newborn lambs and human infants demonstrated tachypnea under heat stress, there is paucity of data in newborn rodents. The decrease in ${\dot{V}}_{E}$with heat stress under normoxic baseline conditions in our study might have resulted from an initial hyperpnea leading to hypocarbia thus suppressing respiratory drive or secondly, from dehydration resulting from high ambient temperature that may override thermal tachypnea (Mortola and Maskrey 2011). Furthermore, breathing frequency was more variable during heat stress, which might have resulted from minimal chemical drive as observed in normoxia and not hypoxia (Cameron et al. 2000; Cummings and Frappell 2009). The increased hypoxic‐induced ventilation gain under high ambient temperature in this study may be the result of an increased sensitivity to hypoxia under high temperature conditions (Petersen and Vejby‐Christensen 1977; Tomimatsu et al. 2003; Chu et al. 2007; Mortola and Maskrey 2011). Indeed, hyperthermia has been shown to reduce autoresuscitation in response to anoxia in rat pups (Serdarevich and Fewell 1999). We demonstrate a posthypoxic ventilatory decline at T_a_ of 33°C that was not observed at T_a_38°C. The posthypoxic decline under thermoneutral conditions likely represents similar mechanisms that mediate the inhibitory arm of the biphasic hypoxic ventilatory response (Coles and Dick 1996; Teppema and Dahan 2010). On the contrary, the absence of the posthypoxic decline under high T_a_ could be from a change in the magnitude or time course of the hypoxic response or a complex interaction between peripheral and central respiratory control that may involve inhibited adrenergic signaling in the pontine A5 region which have been implicated in the control of posthypoxic ventilatory decline (Bach et al. 1999; Schlenker and Prestbo 2003).

While we found no change in HR during normoxic breathing at higher temperature, there was an interaction with hypoxia to increase HR compared to control temperature. The increase in HR with higher temperature is consistent with other studies conducted in the neonatal period of rat and mice (Serdarevich and Fewell 1999; Hall et al. 2001). Warming during hypoxia may cause a decrease in peripheral vascular resistance and mild hypotension stimulating an increase in cardiac output (Seifert et al. 2006) and may also stimulate breathing, thus further increasing sympathetic drive.

### Summary

In summary, we provide an extensive cytokine profile in response to LPS and heat stress in neonatal rats. Furthermore, we demonstrate that LPS administration blunts the neonatal cardiovascular responses to hypoxia which could further compromise oxygen delivery in infants with sepsis. We also report that although LPS administration increases proinflammatory cytokines, it does not lead to fever in 1‐week‐old pups. Heat stress suppressed minute ventilation during normoxia, whereas in combination with hypoxia, a pronounced stimulation of both breathing and HR was observed. Our study reinforces the importance of maintaining the correct ambient temperature for infants and adherence to the safe sleep environment to minimize physiological stress, particularly hypoxia (Horne et al. 2015). This study supports a role for inflammation in the destabilization of cardiorespiratory responses during early life.

## Conflict of Interest

None declared.
